# Supplementary material for: Scheimpflug Tomographic Indices for Classifying Normal, Down Syndrome and Clinical Keratoconus in Pediatric Patients
Source: Diagnostics (Basel). 2024 Sep 2;14(17):1932. doi: 10.3390/diagnostics14171932 (PMC11394033; doi:10.3390/diagnostics14171932)
Supplement: Supplementary file 1 [file diagnostics-14-01932-s001.zip › diagnostics-3148741-supplementary.pdf]

Supplemental Table S1. The values of the all indices for KC and FK discrimination and their diagnostic accuracy.

	KC vs Control				KC vs DS				FK vs DS			
	CUTOFF	SN	SP	AUC	CUTOFF	SN	SP	AUC	CUTOFF	SN	SP	AUC
**TP**	523.5	87.9%	83.9%	0.919	495	59.1%	42.9%	0.524	511	83.7%	71.4%	0.779
**Km**	44.3	80.3%	95.7%	0.895	44.7	75.8%	40.8%	0.532	43.8	89.8%	78.6%	0.88
**Kmax**	45.45	93.9%	90.8%	0.984	47.2	90.9%	44.9%	0.812	44.8	91.8%	71.4%	0.835
**VOL**	63.1	92.4%	29.8%	0.693	59.2	42.4%	95.9%	0.722	57.2	67.3%	85.7%	0.792
**ACD**	3.96	75.8%	27..7%	0.516	3.74	51.5%	77.6%	0.654	3.34	71.4%	57.1%	0.55
**Q**	0.56	84.8%	97.9%	0.925	0.45	92.4%	30.6%	0.808	0.33	91.8%	57.1%	0.722
**AE**	4.5	97.0%	94.3%	0.991	8	84.8%	95.7%	0.946	6	27.7%	100%	0.557
**PE**	9.5	92.4%	92%	0.981	12	90.9%	85.1%	0.947	9	38.3%	78.6%	0.519
**PPI- Avg**	1.135	97.0%	95.4%	0.974	1.19	95.5%	89.4%	0.961	0.92	53.2%	92.9%	0.715
**PPI-Max**	1.435	95.5%	92%	0.972	1.66	92.4%	89.4%	0.938	1.1	27.7%	100%	0.638
**ART-Max**	358	97%	93.1%	0.972	292	92.4%	85.1%	0.916	398	38.3%	78.6%	0.549
**BAD-D**	1.74	97%	96.6%	0.982	2.71	95.5%	85.7%	0.941	0.95	89.8%	35.7%	0.587
**IS**	0.715	88.2%	87.3%	0.928	0.59	92.2%	49%	0.874	1.08	83.7%	50%	0.662
**IVA**	0.225	93.9%	92%	0.965	0.25	92.4%	75%	0.943	0.18	60.4%	57.1%	0.542
**ISV**	23.5	97%	95.4%	0.997	34	90.9%	75.5%	0.923	22	77.6%	57.1%	0.732
**Z3 -1**	0.191	83.3%	82.8%	0.891	0.137	87.9%	44.9%	0.845	0.161	53.1%	78.6%	0.659
**Z3 -3**	0.108	77.3%	77%	0.812	0.075	81.8%	28.6%	0.624	0.086	71.4%	92.9%	0.784
**ALL TOT**	1.913	98%	96.4%	0.999	3.165	90.2%	77.6%	0.931	1.348	89.8%	12.5%	0.548
**ALL HOA**	0.616	98%	96.4%	0.995	0.712	90.2%	75.5%	0.93	0.467	79.6%	62.5%	0.685
**ANT TOT**	2.261	100%	98.2%	1	3.578	92.2%	87.2%	0.949	3.394	78.7%	25%	0.553
**ANT HOA**	0.55	98%	96.4%	0.998	0.834	92.2%	91.5%	0.953	0.449	83%	37.5%	0.606
**POST TOT**	0.94	92.2%	90.9%	0.973	1.001	90.2%	74.5%	0.932	0.956	70.2%	62.5%	0.652
**POST HOA**	0.207	94.1%	92.7%	0.966	0.245	90.2%	57.4%	0.871	0.197	78.7%	37.5%	0.503

KC, keratoconus; DS, Down syndrome; FK, forme fruste keratoconus; SN: sensitivity; SP: specificity; AUC: area under the receiver operating characteristic curve; TP: corneal thickness at the thinnest point; Km: mean curvature power of the cornea; Kmax: maximum simulated keratometry; VOL: corneal volume; ACD: anterior chamber depth; Q: corneal asphericity; AE: anterior elevation from the best-fit sphere; PE: posterior elevation from the best-fit sphere; PPI-Avg: average pachymetric progression index; PPI-Max: maximum pachymetric progression index; ART-Max: Ambrosio's relational thickness maximum; BAD-D: Belin/Ambrosio enhanced ectasia total derivation value; IS: inferior-superior index; IVA: index of vertical asymmetry; ISV: index of surface variance; Z3 -1: third-order Zernike polynomials of vertical coma; Z3 -3: third-order oblique trefoil; TOT: total aberration; HOA: high-order aberration of the entire (ALL), anterior (ANT), and posterior (POST) cornea expressed as root mean square (RMS) data.
